# Reading, Trauma and Literary Caregiving 1914-1918: Helen Mary Gaskell and the War Library

**DOI:** 10.1007/s10912-018-9513-5

**Published:** 2018-03-28

**Authors:** Sara Haslam

**Affiliations:** Department of English, Faculty of Arts and Social Science (FASS), The Open University, Walton Hall, Milton Keynes, MK7 6AA UK

**Keywords:** First world war, Reading, Trauma, Literary caregiving, Helen Mary Gaskell, War library

## Abstract

This article is about the relationship between reading, trauma and responsive literary caregiving in Britain during the First World War. Its analysis of two little-known documents describing the history of the War Library, begun by Helen Mary Gaskell in 1914, exposes a gap in the scholarship of war-time reading; generates a new narrative of "how," "when," and "why" books went to war; and foregrounds gender in its analysis of the historiography. The Library of Congress's T. W. Koch discovered Gaskell's ground-breaking work in 1917 and reported its successes to the American Library Association. The British Times also covered Gaskell's library, yet researchers working on reading during the war have routinely neglected her distinct model and method, skewing the research base on war-time reading and its association with trauma and caregiving. In the article's second half, a literary case study of a popular war novel demonstrates the extent of the "bitter cry for books." The success of Gaskell's intervention is examined alongside H. G. Wells's representation of textual healing. Reading is shown to offer sick, traumatized and recovering combatants emotional and psychological caregiving in ways that she could not always have predicted and that are not visible in the literary/historical record.

In July 1937, the *Book Trolley,* the magazine of the guild of hospital librarians, published a brief history of libraries in hospitals. Owing to the writer’s (self-confessed) advanced age and poor health, the piece was shorter than it might have been. Mrs. Gaskell, C.B.E., knew that in different circumstances she could have written “a good deal on the Past.” But she welcomed the opportunity to provide an account of the “difficulties and influences” under which libraries in hospitals had begun, and she returned to two international conflicts to do so: the Boer War and the First World War (1937, 203). The piece she produced was indeed short, numbering only four pages. However, in 1918 Gaskell had written a longer and more specific version of her history. *The Red Cross and Order of St John War Library* was published by the Red Cross in 1918 and provided a detailed account of the establishment and operation of the War Library which she herself founded in 1914.

This article is about Helen Mary Gaskell’s War Library and the model of literary caregiving on which it was founded – and so it is also concerned with the early history of what would become known as bibliotherapy.^1^ Gaskell chose a quotation from *Titus Andronicus* to head her 1918 pamphlet: “Take choice of all my Library, and so beguile thy sorrow” (1918, 1). The second half of this article presents a case study which demonstrates, using contemporary evidence, the ways in which books did help to “beguile sorrow,” having placed Gaskell and her library accurately in the history of reading during the First World War.

This scholarship, a currently vibrant area of book history, mostly adopts the analytical framework first established by Robert Darnton in 1986. We now know more about the “what,” “where,” and “how” of reading during the conflict than ever before (Darnton 2014, 165).^2^ We also know more about the “who” – as the historic focus on élite readers (as an extension from élite writers) has been challenged by more inclusive, broader assessments (Sutcliffe 2016, King 2014b, Jaillant 2011). The “why” of reading at war is a prominent theme in the scholarship (King 2014b, 375-6), commanding attention through contemporary accounts of felt need for the curative powers of literature: “the bitter cry for books” (Rhys 1916, 1000). Much remains unexplored in this field, however. The American librarian Theodore Wesley Koch’s work on reading during the war, well-mined for data and anecdote (Towheed and King 2015, Laugesen 2012, Sutcliffe 2016, Young 1981), was, for example, dependent on Gaskell’s knowledge and experience in ways that remain unaddressed and which emphasise the importance of reading to those who were sick. Attention to Gaskell and her War Library means that the “how” and the “why” of reading at war can be re-interrogated and newly historicized; her war-time literary caregiving dated back to the Boer War.

My case-study analysis of a First World War bestseller demonstrates literary caregiving in action and, at the same time, illustrates a new version of what I call the “literature plot.” Derived in part from Gaskell’s documents, this version challenges the conventional wisdom about when literature entered Britain’s war and its purpose as conceived by those agents who put it there. I argue that this original purpose was indistinguishable from therapeutic notions of emotional and psychological caregiving and that this was reflected both by the representation of reading in H. G. Wells’s *Mr Britling Sees it Through* (1916) and in accounts of war-time, combatant consumption of that novel. Readers knew that they were deep in books for reasons associated with caregiving – of self or of others – in ways that I also suggest are gendered. Ben Shepherd’s history of military psychiatry emphasises the fact that most soldiers on the Western Front were in their teens and early twenties: “half men, half boys” who may have been in their physical prime but were some way off emotional maturity.^3^ Michael Roper’s exploration of emotional survival in those (he uses the same striking phrase) “half men half boys” (2009, 251) highlights not only their youth but also the importance of mothers to that survival (Loughran 2010). But Roper omits a crucial carrier of caregiving from war-time mothers to their soldier sons: books.

My version of the gendered, emotional-health inflected version of the literature plot is also, relatively speaking, politically neutral – unlike the one C.F.G. Masterman set in motion when he summoned the biggest names in contemporary British writing to a secret meeting to learn how they could contribute to the war effort. It is thus more congruent with the overall public mood in August 1914 as Adrian Gregory describes it than with the official attempt to harness literature’s power for propaganda. Gregory acknowledges a “rather pro-interventionist popular attitude” but argues that the “major organised manifestations of public opinion were pro-neutrality and anti-war […].” In organised feminism, for example, “there was an unprecedentedly broad response in favour of neutrality” (2008 15, 23). Gregory’s revisionist account is important: “[t]he evidence for mass enthusiasm at the time [war was declared] is weak” (11). The concurrent “urge to do something,” which Gregory describes as characteristic of the period after August 4 (35), and which was shown in a pre-war “upsurge in mutual aid bodies, clubs and associations” as well as in “charitable endeavours” in Britain (Grant 2014, 11, 17) was, however, strong. In the case of the War Library, this urge was quickly and non-jingoistically discharged by the seeking and giving of books on a massive and unprecedented scale.^4^

Finally, I aim to encourage with this article the re-balancing of the growing body of research on this topic. There is a less formal, less institutional story to be told here, complementing, for example, Reznick’s focus on caregiving by military and voluntary-aid authorities (2004) or Michael Snape’s on church organizations (2009). Many contemporary readers and writers provide rich encouragement to tell it. And, in contrast to Sutcliffe (2016), I argue that leisure provision, educational opportunity and overt (or covert) moral inculcation, while significant ways of exploring the drive for literary provision from the top down (Liebich 2015), do not get close to the heart of the matter as experienced by readers themselves or as appreciated by one of the most astute authors of the time: the debate about what soldiers ought to read as opposed to what they wanted to read was pithily summarized in a piece on the Prisoner of War-focused Camps’ Library at the end of the war (“The Camps’ Library” 1918). Gaskell’s immediate and empathic response on 4 August 1914 showed that she did get close to the heart of that matter. More significantly still, she was in a position to act on what she knew books could mean at war. (“Dear readers,” she wrote of her library, “this flow of comfort … must not be checked for lack of money, literature or labour” [8].) She takes her place in the burgeoning tradition of organised female empathy in the early years of the twentieth century (Hammond 2014, 31, 121).

## The founding of the war library

In January 1917, three months before the United States’ declaration of war against Germany, Theodore Wesley Koch had been sent by Herbert Putnam, the Librarian of Congress in Washington, on a “special mission” to England. He got sick in the winter weather. As he recovered, he read. He was given a magazine published by internees at Ruhleben internment camp and became fascinated by their reading experiences, especially by “references to a scheme for supplying books to British Prisoners of War” (1918, preface).^5^ He then heard about the “wonderful work” Gaskell had been doing for the sick and wounded through the War Library but suffered a relapse before writing up her story. Arthur P. Young records that the paper Koch did eventually manage to write was read at a Louisville conference of the American Library Association on June 22 1917 and that Board members present heard about the four agencies via which books and magazines were sent to British troops: “the British Red Cross and Order of St John War Library; the Camps Library; the YMCA; and the British Prisoner of War Book Scheme” (12).^6^ Sick, reading, convalescing, Koch’s mind was pre-emptively alive to the significance books might soon have in the States because of what he was learning about what they had come to mean in Britain. Gaskell’s (by then re-named) War Library was first on Koch’s list, but its foundational and wide-reaching significance is nowhere recognised by book history scholars. This omission, which has skewed the resultant research on reading at war, especially with respect to literary caregiving, has persisted despite the fact that *The Times*, for example, attributed in 1915 the “excellent idea of a War Library for hospitals at home and abroad” to Gaskell in its series on the war (*The Times* 1915, 502).

Helen Mary Gaskell, known as May, was born a Melville. Her husband, Henry Brooks Gaskell, had inherited a fortune from his father. The couple took over Kiddington Hall, Woodstock, Oxfordshire, in 1889 but still spent time at No. 3, Marble Arch, their London address from 1890 until 1907. Gaskell published her account of the beginnings of the War Library in her 1918 pamphlet:

Surely many of us lay awake the night after the declaration of War, debating the question how best we could help in the coming struggle […]. Into the mind of the writer came, like a flash, the necessity of providing literature for the sick and wounded. The same evening four of five friends dined together and talked the idea into shape […]. (1)

The dramatic force of her concept, a war library supplying sick and wounded soldiers and sailors stocked through an appeal for donated books, was compounded by the social and political freight of the friends she was with that night. Herbert Asquith was among the other guests at the house party. The support of further prominent members of the political and social establishment was immediately sought. Lady Battersea and Lord Haldane,^7^ along with Sir Arthur Sloggett, then head of the Royal Army Medical Corps, were crucial in this respect, as was Gaskell’s brother, Mr. Beresford Melville. “Haldane secured the recognition of the War Office” for the library, explained *The Times*’s “History of the War” (1915, 502), while Sybil (Countess) Brassey, known for her suffragist beliefs, and Lady Battersea both joined the War Library Committee. Brassey noted in her autobiography that one of Gaskell’s first actions was to telegraph to Lady Battersea “for the loan of her London home [Surrey House, a mansion then empty, situated at Marble Arch], and Lady Battersea wired in reply, ‘[c]ertainly, with pleasure’” (Brassey 1923, 184).^8^ Within days the committee had this central location to store donated books. Alfred (Lord) Milner, described as “the most important personality in the Government” from 1916-1918,^9^ had been a friend of Gaskell’s for some years – the first letter his biographer quotes dated back to 1893 (Wrench 1958, 146). The couple had dinner on 5 August, and he joined her committee as well. Viscount Samuel was an equally important supporter. Though not a member of H.M.’s Government when he initially became involved, he was Postmaster-General. His memoir details his pleasure at heading up the department responsible for administering the War Library’s national collections and international deliveries, experience he took with him to the post of Home Secretary on January 1, 1916 (Samuel 1945, 109-110). Gaskell recorded the visit he made to the front where he “saw the need of literature for the men” and was converted to their cause (3).

Until the War Library was affiliated with the Red Cross in 1915, Gaskell and Melville ran it out of their own pockets with some additional financial assistance from their friends including Brassey who took on the library’s work in Alexandria later in the war (185). A budget sheet held in the Red Cross Archives shows cash donations between April and October 1915 of £597.18.3, of which £20.00 was refunded “to Mrs. Gaskell for expenses incurred.” The sheet records that the library was paying wages at this point (£56.0.11½), but the largest expense related, predictably, to the “carriage of books and parcel post” (£88.3.3½) (JCO/6/3). Gaskell had appealed to Dr Hagberg Wright (Secretary and Librarian of the London Library) for his help the previous autumn when the volume of donated books arriving after the first public requests became unmanageble. Wright brought some of his staff with him when he answered her call. He became Honorary Secretary of the War Library, alongside Gaskell. The next budget sheet, detailing receipts and expenditure in November 1, 1915 to October 31, 1916 shows the new name of the library (“British Red Cross and Order of St John War Library”) and records a vastly increased budget, including £2800 in grants and £270 in donations (JCO/6/3).^10^

The Red Cross was a logical organisational and ethical system with which to affiliate, as book donations increased still further across 1915 with one historian of medicine has noting its “civilizing mission” in the war (Cooter 1993, 1558). There were already links between personnel: Milner was Vice President of the Red Cross’s County of London Branch and had been since 1914. The *Oxfordshire Branch History* of the British Red Cross notes a “Mrs Gaskell, Kiddington Hall, Kiddington,” as registered with the Red Cross in 1911 and names her “commandant” of the “Women’s Detachment.” In the autumn of 1915, Gaskell and Wright approached the Joint War Committee of the Red Cross and St John to request affiliation. This the Committee “most kindly consented to do,” assuming financial responsibility in return for the War Library’s undertaking to “supply the literature they and their Hospitals require” (Gaskell 1918, 3). “We are most proud to be a branch,” Gaskell wrote in response.

Soon after, the library applied for registration as a Charity under the War Charities Act, 1916. Related papers are held in the London Metropolitan Archives as part of the London County Council archive. In the first application, Lady Battersea’s London mansion, Surrey House, was named as the administrative centre of the charity, while Gaskell, Melville (the original treasurer), Hagberg Wright, Lady Battersea, Milner and Brassey had been joined on the Committee by Mr Alfred Keyser as Honorary Treasurer, the Countess of Lucan, and John Bailey. By 1916, Gaskell’s library was thoroughly well-supported and well-connected in terms of its management and organisational structure: it could mobilize her vision of literary caregiving from a politically experienced and well-funded base. Industry supported it, too. Publishers provided books at trade prices and also made gifts to the venture (*Reports of the British Red Cross Society* 1921, 267).

In a later application for registration under the War Charities Act, the “precise objects of the Charity” are noted as “[t]o supply free Books and Magazines to Naval Military & Civil Hospitals and Hospital Ships and Homes and similar institutions for the care of sick or wounded within the United Kingdom (but more especially to hospitals, hospital ships homes or institutions treating or having the care of persons injured in the war).” The form of words changed slightly over time, but the central notion, of acquiring donated books to supply free for the care of those sick and wounded while serving, was constant after Gaskell’s 4 August self-described epiphany. Gaskell’s biographer further explains that epiphany through reference to her family’s earlier experience of war.

In May 1900, May’s son-in-law had been wounded at the Siege of Mafeking. “On hearing the news,” Josceline Dimbleby writes, “May, who had recently gone through an illness which she said ‘was made bearable by books,’ suddenly knew what she must do:” she sent some books. Her son-in-law, Lionel, replied in gratitude for this gesture, explaining its effect: “[i]f I lived to be a thousand no words could ever tell you what your books are to us in the ward. We have cut up the Rudyard Kipling volumes into numbered parts, and we pass them down the beds, for a volume each is too precious.” “I never forgot this,” May said afterwards (Dimbleby 2004, 179-80).^11^ Fourteen years later that act, originally performed out of self-care and extended with empathy to a wounded family member far from home, was re-imagined and re-scaled in a new time of peril. It was an act calculated to make connections (Gaskell believed in using the “personal touch” with patients in Hospital [5]) and to fill an emotional vacuum. “What was our astonishment when not only parcels and boxes, but whole libraries poured in,” Gaskell remembered (1). The War Library tapped so successfully into the public “urge to do something” that very soon the Marble Arch mansion was necessary to house all the books. High points of its activity included the packing of six thousand books and magazines for a Hospital Ship in less than twenty-four hours. But its caregiving roots remained intensely personal: “workers made a point of keeping in touch with an individual patient making a special request for a certain class of book” (*Reports of the British Red Cross Society* 1921, 271), even as Gaskell acknowledged the enormous demands: “when Gallipoli was filling every bed with sick, cables would come, ‘[s]end 25,000 books at once’… when perhaps the day before Malta had called for 10,000” (4).

The novelist and journalist H.G. Wells was at C.F.G. Masterman’s famous meeting on September 2, 1914 at the new War Propaganda Bureau. Masterman’s aim, as is well-known, was to “conscript” the “Edwardian literary establishment” to his propaganda drive (Hynes 1992, 26; Buitenhuis 1988). But, as the history of the War Library demonstrates, September 2 was late. Literary power had already been deployed through what Koch calls in his account of Gaskell’s work “the first appeal of the War” (1917, 5), an appeal that generated a relationship between books and therapy at war that would eventually see millions of texts changing hands.

## Analysing Gaskell’s contribution: Publishing, ‘book hunger,’ and fiction at war

On August 8, 1914 the novelist Ford Madox Ford wrote of the war with Germany that “whichever side wins in the end – my own heart is certain to be mangled in either case” (1999, 208). At the same time he distrusted both jingoism and the newspapers, while his friend Wells’s journalism helped shape aspects of the popular patriotic mood. “I am radiant this morning,” Wells wrote to Rebecca West on 5 August. That radiance was caused by the birth of their son, Anthony, the day before and the outbreak of war. “I have a manchild in the world – of yours. I will get the world tidy for him” (Hammond 1991, 85). Wells’s “intense patriotism” may have “caught the mood of the nation” (Hammond 1991, 85), but soon he was focused on ideas of a World Congress to enforce international law, while Ford was writing the poem, “Antwerp,” as a result of witnessing Belgian refugees’ arrival in London.

Like Ford, who asked as early as 15 August “[w]hat then will be the future of the arts when we have a little quiet again?” (Ford 1999, 208), Wells was immediately concerned about the effect of the war on the arts in general and on literature in particular: “[w]ar is just the killing of things and the smashing of things. And when it is all over, then literature and civilization will have to begin again” ([Wells] 1915, 284). His fear was personally profound, as well as professionally driven. At age seven, and again at eleven, Wells read his way through illness, later emphasising the part books had played in recovery as he developed his autobiographical story. His “mind was born anew,” as a result of his sick-bed reading, he wrote as he reflected on the role literature had played in his young life (Hammond 1979, 5, 11).

Wells’s particular concerns about the effects of war on readers are demonstrated in his fiction, and his fears about the war-time health of the arts were widely shared. Jane Potter has summarised them from the market perspective: “paper shortages and a loss of staff to the Forces were coupled [in publishers] with an anxiety over the reading public’s readiness to exercise its spending power” (2008, 52). But as early as November 1914, publisher and author Arthur Waugh could confidently assert that the impact on the UK publishing business was not going to be as disastrous as had been predicted (1914, 766). In fact, it soon became plain that literature was thriving in ways that confounded all expectation (Potter 2008, 54). I am most interested in that phenomenon, and the reasons for it, when it occurred beyond the market boundaries (where books changed hands for nothing). The War Library is a too-little investigated route to consumption and was at the time an increasingly urgent focus of need.

“The Power of the Book […] was never felt as it is now,” wrote Ernest Rhys, editor of the Everymans Library, in May, 1917. “[A]n extraordinary vast new audience has appeared in camp and hut, such as no-one can have quite foreseen when the great disruption began” (*Red Triangle* 123 (3), 442). A few months earlier, in October 1916, he observed in the same publication how “the last thing Marlborough or Wellington would have thought an army wanted was books to read,” but that in this respect “our army is like no older one that ever took the field” (94 (2), 1000). He was right in one sense. As already noted, publishers had expected demand to fall when war came. Fewer new books were published in Great Britain year on year between 1913 (a total of 8625) and 1917 (5716). Demand itself, however, went up, and so, accordingly, did prices of second-hand books (Ellis 1975, 30-2).

Koch reported in 1917, after his research trip to investigate the British war library service, that English booksellers were experiencing a famine of seven-penny and shilling books because of the scale of demand from the trenches (1917, 3). Furthermore, he recorded that the YMCA’s *Red Triangle* library had sent 83,640 books and magazines in the previous five months to a combination of destinations, including the home camps, France, and overseas bases (3-4). More than 30,000 books had gone to Salisbury Plain when it was known that Colonial troops were about to mass there. Edmund King has shown that it is crucial to understand that these were not just collections of poetry and Shakespeare going to an officer class, as suggested by Fussell in *The Great War and Modern Memory*, but a huge range of material, finding an equally wide range of readership. King makes this point in an article on E.W. Hornung, Conan Doyle’s less famous author-relative, and the “day to day operation” of a First World War soldiers’ library for which Hornung was responsible. King quotes evidence supporting Koch’s assessment of the cheap editions’ famine (2014b, 363-4), while Rhys described the inability of those who did not fight to comprehend the effect of that “book-hunger” on the man who is “cut off” from books in his article in October 1916 (1000).

Capitalism certainly profited from the war’s effects on working patterns and attachment structures combined. Rather than interpreting the lessons in this fact from a Marxist perspective, however, I suggest that literature was increasingly operating, and being therapeutically circulated, in what I described earlier as a collective emotional vacuum – of the most disturbed kind. Gaskell anticipated both the vacuum and literature’s effect on it although she did not predict the scale in either case. “People were urged to give something that they themselves really cared for,” records Koch of the War Library appeals (1917, 14); the gift itself, thus invested, would carry and communicate the care as part of its felt effect. And, as Gaskell herself recorded, the appeal elicited a totally unexpected response from the donating public who evidently cared a great deal. The Red Cross archives state that traffic outside Surrey House was regularly brought to a standstill by deliveries. One individual donated a library of 35,000 books.^12^ The Queen herself showed an interest in the Library in 1915 as *The Times* reported on 4 December. The scrapbooks mentioned in the article were so-called because Kipling had suggested them – brightly coloured large-format sewn-together books full of pictures, and jokes or anecdotes, pasted in by volunteers (Koch 1917, 7).^13^

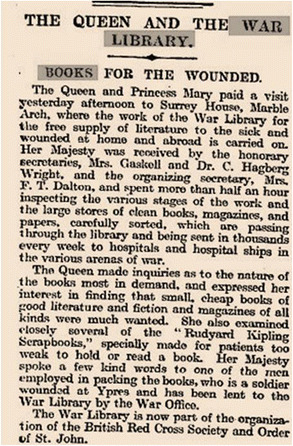



Gaskell’s effective omission from the historiography since book historians post-Darnton began to work on reading during the war (Ronald Sturt and Antonia Bunch both cited her in their work on hospital libraries in the 1970s) has meant that her therapeutic literary interventions and their relationship to the tremendous surge of public investment in literature at the outset of war have been similarly invisible. Relevant contextual and explanatory detail that would have dated her interventions to a previous international conflict, also absent, further compromise the extant scholarly narrative. People wanted to donate books to a venture conceived primarily for soldiers’ comfort and not for their instruction: those omissions therefore compromise understanding of the “when,” the “where,” the “how,” and particularly the “why” of reading during the conflict. The “buy-in” of the British public to the therapeutic project was as extraordinary as the levels of need that public helped to meet. And yet Laugesen, for example, mistakenly names Lady Battersea as founder of the War Library (2012, 15). More commonly, Grant’s elision of the formation of the War Library and the later Camps Library provides the basis of the narrative, and he credits Sir Edward Ward with founding both on Kitchener’s request: “[o]ne idea Ward had was to ensure a supply of books and magazines for the camps and billets” (2014, 129; and see Ellis 1975, 47). There are no references to support Grant’s assertion that Kitchener asked Ward to “look after the welfare of the men” in this way. Sutcliffe cites Grant and calls the camp libraries, which largely served prisoners of war, “Kitchener’s initiatives.” She names Gaskell as the secretary, working “under the guidance of Hagberg Wright” (2016, 5-8), despite the documentary evidence from the Red Cross naming Gaskell both as “Honorary Secretary” and a Director of the War Library (*Reports of the British Red Cross Society* 1921, 9, 267). Gaskell’s own account of the Camps’ Library’s beginnings was given in 1918: “Dr Hagberg Wright, Mrs Anstruther, Sir Edward Ward and I met and discussed the divisions of our labours, as the field of work was increasing so largely….” (3).

Gaskell also records that it was she who, faced with extraordinary numbers of donated books, appealed to the “most capable and kind-hearted librarian” (2), and Hagberg Wright published a letter in *The Times* on 22 August 1929 in which he identified her sole organisation in the early days of the war. Wright had also written to the editor of *The Times* to correct the record regarding the War Library’s relationship with the Camps’ Library (1 November, 1916). The *Reports of the Joint War Committee and the Joint War Finance Committee of the British Red Cross*, published in 1921, state that Hagberg Wright “voluntarily assisted the War Library” with five of his staff after his co-operation had been sought and that “in November, 1914, after consultation with the War Library, Mrs Anstruther and Sir Edward Ward founded the Camps Library” (267), corroborating Gaskell’s account. “The Red Cross War Library” section of the *Reports* ends as follows: “we cannot conclude this brief account of one of the most interesting of the efforts made by the Red Cross without a warm acknowledgement of Mrs. Gaskell’s untiring labours, and of the assistance and advice given by Dr. Hagberg Wright” (272).

It is crucial to an understanding of the “why” of books at war to appreciate that, like Koch’s later written polemic, Gaskell’s responsive act was borne originally of both empathy and sickness. The relationship between literature and caregiving was primary and integral in the face of war. The results of the Library’s “first appeal of the War” and the way it which it was managed both illustrated and strengthened the bonds of this relationship, as did the most avidly-consumed texts themselves.

A later appeal in *The Times* asking for increased donations on 24 September 1917 (“Books for Soldiers”) reported that 10,000,000 units had been sent prior to that date. But even these numbers could not satisfy demand. And so the final necessary element of the context for thinking about literature and caregiving at war before turning to what writers understood of it is the level of need among British soldiers, some of which was met, much evidently not. “Sir,” wrote Gaskell and Habsberg Wright in July 1916 to *The Times*, “The wounded are pouring into our hospitals, where the need for literature exceeds all previous needs. We require an enormous and immediate supply….” (“Books for the Wounded”). *The Times* and other newspapers carried new appeals, but “demand far exceeds supply” reported Koch. “Men feel,” he wrote in 1917, “that if they had twice as many books as at present they should not have enough” (15).

Gaskell’s plea to the public to donate what was “dear” (1918, 8) to the men (Kipling, Nat Gould, O. Henry, Marie Corelli), rather than privileging examples of, as the Red Cross *Reports* explained it, “a higher class of literature” (271), as well as Koch’s account of how men would hide books for fear of them being taken away, or of how books were “literally read to pieces” (1918, 82) are the most pertinent criteria as I turn to explore Wells’s practical appreciation of literary caregiving at war. His fiction offers a rich variety of ways of interpreting the felt need for books that Gaskell had anticipated as war broke out.

### Mr Britling

*Mr Britling*, by H. G. Wells, was not, to quote Ivor Gurney writing home from the trenches desperate for something to read, “any sort of book” (Gurney 1991, 119). Translated into Danish in 1916 and into French, Swedish and German the following year, it was one of the best-selling titles of the war and perhaps the “most popular” novel (Gregory 2008, 153). Its prominence in the *Red Triangle*’s “Leisurely Letters” column contributed to its ubiquity.^14^ It was first featured there in October 1916, when a review noted that Wells had “discovered something more wonderful than an Invisible Man or a Food of the Gods” in his most recent protagonist (*Red Triangle* 93 vol. 2, 987). In April 1917, the columnist has to request his copy of it back from a correspondent, wondering if “A” hasn’t returned it because he’s reading it for a second time (*Red Triangle* 118 vol. 3, 329). The novel had gone through eleven impressions or editions by the end of 1916, enjoying an “extraordinary public response” on the home front, and Wells “was gratified to find himself a best-selling novelist” (Hammond 1979, 181; Hammond 1991, 97). Frank Worsley, a devoted fan, published his *Letters to Mr Britling* in 1917. The foreword described the novel as his “sole literary companion during many hours of many weeks” as a serving Chaplain in France (1917, foreword). Gorky wrote to Wells calling it “the best, most daring, truthful and humane book written in Europe during the course of this accursed war” (Cockrell 2005, 80), and many contemporary soldier-writers and readers cite it in their memoirs or letters. Henry Dundas read the book almost as soon as it came out and “at once” recommended it, recalling how he did so in a later letter (1921, 113-4); a prisoner of war at Augustabad was reading it by February the following year and “liked it immensely – especially the 3^rd^ quarter dealing with Hugh & his enlistment & his letters & his death & B’s grief,” concluding that it was “most beautiful” (Beyfus, unpublished diary). Lieutenant Paul Jones praised the “way in which [Wells] expresses the point of view of the average man,” though he felt the novel was not up to Wells’s “very best” (1918, 228).^15^ Post-war, Ford Madox Ford remembered suffering from insomnia and hearing in the night an officer send an orderly to reclaim a copy of the novel from the Major if he had finished with it (1980, 122).

The extravagant consumption of what was itself a textually obsessed (and highly autobiographical) novel demands investigation. This combination renders it a key text in any analysis of literature and caregiving at war. In the contexts of an emotional vacuum and the “book hunger” experienced by serving soldiers, Wells’s perceptive genius becomes clear: he did not just give those readers a book, he gave them a book which displayed in a range of keenly relevant ways his understanding of why they might need it so badly. The dust jacket of the 1916 Cassell edition stressed the “sympathetic and sensitive intelligence” of its subject. Books at war in general, I suggest, delivered the experience of care to their readers in the ways that May Gaskell intended – by activating the memory of familial affection and familiar routine, distracting and entertaining, demonstrating the gratitude and the comparatively small but significant sacrifices of the donating public or by helping men to cope in other ways. As Reznick puts it, “experiences that played out in the wartime culture of caregiving ... involved rational and familiar processes combining with stark realisations ... of unprecedented circumstances” (2004, 3). Contemporary records demonstrate what that looked like in literary terms: books by Ian Hay, for example, that made readers “feel welded” into a “splendid human mass” or that helped them understand how the “common man deals with the impossible” as Patrick MacGill’s writing did (*Red Triangle* 85 vol. 2, 793; *Red Triangle* 87 vol. 2, 840). *Mr Britling* also delivered literary caregiving in ways that Gaskell did not, perhaps, anticipate but that post-Freudian criticism can help us to understand.

At the outset of the novel, England itself is offered up to the reader as “like travelling in literature” (1916, 6). Encouraged through Mr Direck’s framing vision to experience the country textually, at one remove, as “Washington Irving’s England,” we meet a writerly hero who “sniffed at the heels of reality” and then translates the smell into the written word (10). When Britling is not writing, however, chaos reigns. Washington Irving has not sufficiently prepared Direck for “his first meal in a private household in England” (20). No one is introduced, many people are present, and the “problem of relationship” threatens to “dissipate and consume Direck’s mind entirely” (22) – the scene is a plot in search of a responsible author. Direck summarises his discombobulation: “‘I thought when I looked out of the train this morning that I had come to the England of Washington Irving. I find it is not even the England of Mrs Humphry Ward’” (33). Stage one of readerly engagement with literature in the text is fuelled by humour; it is manufactured via geographically dislocated nostalgia and delayed gratification coupled with conflicted admiration of the novelist’s power.

Later, the approach of war is represented and consumed textually: as “narrative and spectacle” (Frayn 2014, 50). Newspapers, of course, carried this information everywhere from the source of what Wells calls “the colossal crystallising of accumulated antagonisms” (160), but *Britling* is unusual in the amount of its print it devotes to the consumption of the written news. The novel’s authenticity, its generic reach, are enhanced by this technique, as they are by references to the Bryce report, the movements of refugee populations, and the details of battleship manoeuvres and encounters, but it is more than that too because of Wells’s attention to shared textual consumption. Its realism on both counts would have recommended it to soldier-readers (Towheed and King 2015, 12). On the morning of 3 August, Mr Britling cannot be prised from his reading and rereading of the headlines (168), which are merged with accounts of the flower show to which he is being entreated to come, the reality of which does not have him “sniffing” and which will soon be overwhelmed: “then across all the sunshine of this artless festival there appeared, as if it were writing showing through a picture, ‘France Invaded by Germany; Germany Invaded by Russia’” (170-1). The picture is invaded by capitalised print. After the declaration of war, Mrs Britling shares the paper with Mr Britling as they stand by the bed of begonias (189); father and son read the *Observer* together, while it is spread open “on a garden table under the blue cedar” (221). Joint discussion of the news often necessitates the finding of other books in the Britling household, Whitaker’s Almanack, for example, and Webster’s Dictionary (186). Such guides provide additional or corrective information necessary for negotiating the changing world and offer security – as does the *Encyclopaedia Britannica* to Christopher Tietjens’s shell-shocked brain in Ford’s *Parade’s End* (Haslam 2014, 42-3). When Britling comprehends the “flimsiness” of the credit system and that the security of their lives may be over, another book helps him to find his words and to face the new world order. Gissing’s *Veranilda* provides something closer to material solidity than the banks, the house, and whatever between them he and his wife were now “worth” (193). This is the second stage of Wells’s exploration of what books can do for their readers.

The third stage relates to the emotional and psychological significance of reading as a connective and containing act, which grows as reality alters irrevocably and as the ability to write recedes. In *Britling*, when war is declared, we wait to see if Mr Britling can find the same peace that descended as he anticipated writing the “plain common sense of this Irish situation” days earlier, while fingering his familiar fountain pen (121). Can he find similar words borne of the “deep passion of sanity” in the face of which all “squabbling” would cease? As the “threat of death and extinction” is unveiled, he is certainly writing earnestly (182). But over the next hundred pages or so, what transpires is his struggle to make words bring the war home: “it was rare that he really seemed to be seeing the war […] all the time he was at least doing his utmost to see the war, to simplify it and extract the essence of it” (206) and yet “still he could find for himself no real point of contact with it all except the point of his pen […] he was always desiring some more personal and physical participation” (246). Mr Britling’s pen loses its potency, and the novel now, in ways that it has been preparing for in all its staged consumptions of print of various kinds, becomes much more interested in what Mary Jacobus calls, in her book about the process and cultural activity of reading from a psychoanalytic perspective, the “scene of reading.” Britling is given his “point of contact,” and Wells’s text becomes one devoted to the treatment of suffering and emotion in print. His characters, as readers, are shown to be engaged primarily with loss, emotional distance, and managing their effects, just as those who are consuming his text are likely to be.

In her study, Mary Jacobus analyses Matisse’s 1947 depiction of two figures reading (“La Silence habiteé des maisons”) using Winnicott, Klein and Freud to explore the psychological extent of the book as “more-than-object” (1). *Mr Britling*’s displayed understanding of the relationship between literature and caregiving, and some of the reasons for its popularity among fighting men, are, I suggest, tightly bound together amid the complex of ideas suggested by the psychoanalytic approach to the act of reading. “Fiction is,” Keith Oatley has argued, “a continuation of the creative play of childhood, not just for authors but for readers [and] it takes place in what Winnicott called the space-in-between […] originally the space between the infant and mother […]” (2011, 55). Wells’s novel, consumed in such numbers by emotionally-starved and book-hungry contemporaries, offers one of the best textual examples for exploring what encounters with the written word can show readers about need, about absence and presence, and ultimately about love.

Jacobus works closely with James Strachey’s 1930 essay, “Some Unconscious Factors in Reading.” She pays particular attention to his noting that “the discourse of reading is shot through with metaphors of oral consumption” like chewing, for example, and that reading is a way of “eating another person’s words.” (27). Wells’s understanding, conscious or no, was strikingly similar: he may not have been able to theorize it at this point in literary-psychoanalytic history, but he could certainly explore it in fiction. An “age of psychoanalysis” had been applauded by Lou Andreas-Salomé – in Vienna, not London – as early as 1912 (1987, 70), but J. C. Flügel noted in 1933 that “literary men” had initially demonstrated a more “ready acceptance” of its tenets than psychologists or physicians (1933, 286). Immediately after Mr Britling realises his pen has lost its potency and before the novel’s dramatic turning point and new focus on love in words, Wells digresses into a memorable discussion of infantile feeding. The Belgian refugees Mr. Dimple is housing un-nerve him. The man is an “Atheist” and tried to kiss the Hickson girl, but it is the woman’s breasts that have had the greater impact: “And his wife,” Dimple says to Britling, “a great big slow woman – in every way she is – Ample […]. I do so *wish* she would not see fit to sit down and nourish her baby in my poor old bachelor drawing-room – often at the most *unseasonable* times. And – so lavishly….” (258).

The English “bachelor drawing-room” is not the place for the clock-resistant, instinct-driven behaviour of the Belgian outsiders or for the unproblematic apprehension of infant satisfaction through lavish breast-feeding. However, after its gently humorous depiction of Dimple’s repression and barely-disguised envy, the novel immediately experiments with ways of showing more generally experienced (and appropriate) satisfaction – through reading. Melanie Klein argued in an influential essay from 1931 that Strachey had shown that “reading has the unconscious significance of taking knowledge out of the mother’s body” (1991, 241), while books and paper were interpreted by Freud as female symbols as Wells was writing *Mr Britling* (1991, 189, 193). In Wells’s novel, the emotional context for the climactic act of reading – the letters exchanged between father and son – is immeasurably heightened by the fact that Hugh’s mother, Mr. Britling’s first wife, is dead. This is the primary emotional vacuum at the heart of Wells’s text, one that its “scene of reading” both represents and seeks to assuage in its protagonists. Its readers gained a similar opportunity to process their own feelings of loss and trauma: an important reason, I suggest, why “Correspondent A” held onto the novel, why it was found to be “most beautiful,” and why it was a war-time best-seller.

When Hugh first joined the Essex Regiment as a private, the belief was that the war would be over before Hugh was eighteen. Hugh trains hard and is home seldom; and his letters “become a very important influence upon Mr Britling’s thought” (299). He quotes them, treasures them, is proud of them, like countless other parents. In the first letter Hugh writes, when he is bored and cross, his emotion is due to the stupidity and ineffectualness of his officers. His captain “is afraid of printed matter,” and Hugh, alienated and unsupported, pours his heart out in print to his father (303). Soon after, when Hugh falsifies his age and leaves for the front, there is a meeting between father and son that constitutes a textual pause. Hugh describes going to war as leaving a boundaried domestic space, a secure room, to go “outside” (315). Though Mr Britling’s idea of their home is as one “without sides,” open to “novel ideas” (256-7) (about reading, for example), Hugh describes a more intimate and enclosed space, as he gathers himself for leaving it. On separating after their talk, they each go to their rooms. Hugh sleeps; Mr Britling, in his own supposedly secure writing room, does not. But what transpires is that they leave that boundaried space together. What Mr Britling does that night is fall in love with his son, rediscovering him, delighting in his strength, his wisdom, and his beauty. The intensity of the physical reaction is astonishing and calls attention to that other primary absence – the wife and the mother – in the text as this new loss is anticipated: “it was as if he perceived the beauty of youth for the first time in Hugh’s slender, well-balanced body, the delicate pencilled eyebrow that was so like his mother’s” (316). The letters that father and son begin to exchange are freighted with this original absence. Hugh leaves the next morning, and Wells tells us that from that point Mr Britling “watched the post-man like a love sick girl” (320).^16^

Michael Roper opens his book about the war with a harrowing account of wounded men dying, calling for their mothers. He wants to know what “familial and emotional resources” those who did live through it drew on (2009, 4). He argues convincingly that letters from home were crucial but does not explore the psychological importance of reading in and of itself. In one of his letters home, Hugh Britling shows his father how much he wants books: “‘[w]e read of course. But there never could be a library here big enough to keep us going…’” (327). He reports that one of his comrades wants books “you can chew” (327). In this long communication about the soldiers’ need for books, Hugh is showing his faith in words and making a literary, loving connection at the same time. The popularity of Wells’s novel, a novel that confronts death, absence, and repression as well as communicating the love and hope maintained and contained by words on a page, illustrates how necessary the gift given in reading was. That popularity might also demonstrate how, at a more unconscious level, reading offered a way for the pleasure-seeking and still vital infant of the psychoanalytic model (or Roper’s frightened and comfort-seeking “half men half boys”) to receive nourishment and satisfaction – of the kind that could be experienced far away from home by those avid, suffering readers whom May Gaskell also held in mind. Roper cites Klein on the ego of the young infant, constantly moving between states of integration and disintegration: “When anxious, the ego tends towards disintegration” (260). Hugh Britling is unafraid to reveal how, when Jewell is killed, he began to cry, “‘like a baby’” (357). He feels like a “‘scared child,”’ Wells says, as he gives this experience to other soldiers in containing words (358).

## Conclusion

Reznick’s *Healing the Nation* (2004) discusses the main sites where caregiving took place in the war. One of those he focuses on is the YMCA hut (other church organisations ran them, but the YMCA’s were the most famous). Books were a significant element of the caregiving provided in those huts. Hornung was “ministering to soldiers through books,” King suggests, and was also creating a consciously masculinized space to do so: there was a deliberate imitation of the club smoking-room in the hut he ran at Arras (2014b, 369). That may be so, but the wartime “scene of reading” means that the symbolic mother will have been present in that enclosed space, too. Koch reports on a soldier wanting Psalm 23 to read because his mother read it to him every day (1917, 30).

Literature and caregiving were intertwined at war. Wells knew this and used his best-selling novel to explore how and why. The strength of the conceptual and emotional bond between the two was testified to by the need for books at war and the currency of phrases such as “book hunger.” Gaskell’s formation of the War Library and the public response to it, as well as the war-time popularity of *Mr Britling*, all illustrate and help us to understand that bond, I have argued here. The official record, more focused on the role of prose and poetry in propaganda or on top-down accounts of reading at war, has, rather, served to disguise it. Contemporary readers’ self-identified need of books mean that this bond should not be ignored, but it also compromised one famous officer’s ability to do his job:

Now that our intensive training is nearly finished I am easing off a bit and allowing myself to enjoy books. The result is that I immediately lose my grip on soldiering and begin to find everything intolerable except my interest in the welfare of the men. One cannot be a useful officer and a reader of imaginative literature at the same time ([Sassoon] 1936, 223).

Siegfried Sassoon experienced a fundamental opposition between the empathic, connective life in books and the organised destruction of war – in which as an officer he played an active and successful part. Reading imaginative literature and being a “useful” officer at the same time seemed impossible to him. But Sassoon read anyway—Hardy, Conrad, and Shakespeare among others and broke down, of course, as well (Sassoon 1983, 152, 230). Unafraid of print, though evidently conscious of its power, Sassoon wrote later of the transformational experience of caring for his men. Before books were propaganda, they offered a deeply necessary and satisfying bond in a fragmenting world, a fact that was appreciated by May Gaskell and, through her, by Theodore Wesley Koch, readers with significant agency who understood caregiving and would alter the experience of the traumas of war.
